# Quality of referral letters in oral medicine: a two-year study in a Brazilian public dental specialty center

**DOI:** 10.31744/einstein_journal/2026AO2330

**Published:** 2026-08-14

**Authors:** Beatriz Pontim Pistolato, Lorena Gasques Contin, Hugo Sobrinho Bueno, Fernando de Souza Buzo, Saygo Tomo, Luciana Estevam Simonato

**Affiliations:** 1 Universidade Brasil Faculdade de Odontologia Fernandópolis SP Brazil Faculdade de Odontologia, Universidade Brasil, Fernandópolis, SP, Brazil.; 2 Universidade de Campinas Faculdade de Odontologia de Piracicaba Department of Oral Diagnosis Piracicaba SP Brazil Department of Oral Diagnosis, Faculdade de Odontologia de Piracicaba, Universidade de Campinas, Piracicaba, SP, Brazil.; 3 Universidade Brasil Faculdade de Agronomia e Medicina Fernandópolis SP Brazil Faculdade de Agronomia e Medicina, Universidade Brasil, Fernandópolis, SP, Brazil.; 4 Universidade de São Paulo Faculdade de Odontologia São Paulo SP Brazil Faculdade de Odontologia da Universidade de São Paulo, São Paulo, SP, Brazil.; 5 Universidade Brasil Bioengineering and Environmental Sciences Programs Fernandópolis SP Brazil Bioengineering and Environmental Sciences Programs, Universidade Brasil, Fernandópolis, SP, Brazil.; 6 Centro de Especialidades Odontológicas Dentistry, Oral Medicine Fernandópolis SP Brazil Dentistry, Oral Medicine, Centro de Especialidades Odontológicas, Fernandópolis, SP, Brazil.

**Keywords:** Oral medicine, Referral and consultation, Primary health care, Communication, Mouth neoplasms

## Abstract

**Objective:**

In the Brazilian public health system, referral letters, issued by general dentists and physicians who perform initial clinical assessments, is the main source of information in arriving at a final diagnosis. However, the consistency and completeness of the referral letters remain poorly explored. Therefore, we assessed the quality of referral letters received by an oral medicine service at a dental specialty center over a 2-year period (2023–2024) and characterized the diagnostic profile of referred cases.

**Methods:**

This census-based retrospective cross-sectional study analyzed 430 referral letters using 8 predefined criteria (0–8 points) on lesion characteristics, clinical history, and diagnostic hypothesis. The referral letters were classified as poor (0–1), regular (2–3), good (4–6), or excellent (7–8).

**Results:**

All referrals were from public services; 47.0% were issued in 2023 and 53.0% in 2024. Referrals to dentists accounted for 97.2% of the referrals. Most letters were rated as regular (60.5%) or good (23.0%), while 15.6% were classified as poor and only 0.9% as excellent. Frequently reported descriptors included lesion location (84.2%) and primary morphology (76.5%). Clinically relevant details such as symptomatology (22.8%), time of evolution (16.0%), lesion size (3.5%), and limits (2.3%) were mostly absent. Diagnoses included benign lesions (71.9%), potentially malignant disorders (10.7%), normality variations (6.3%), and malignant lesions (3.7%); 7.4% of the referrals had no diagnosis. Referral quality scores differed significantly across diagnostic categories (p=0.0076), with lower scores observed in cases without a final diagnosis.

**Conclusions:**

Despite the predominance of benign lesions, more than one in every seven referrals, which were often incompletely documented, involved potentially malignant disorders or malignant lesions. Strengthening referral documentation through targeted training and standardized electronic templates may improve screening accuracy, support the earlier detection of oral lesions, and reduce diagnostic delays within the Brazilian unified health system.

## Highlights

■Most referral letters lacked essential clinical information.

■Lesion size and limits were rarely documented in referrals.

■High-risk oral lesions often had incomplete documentation.

■Standardized templates may improve early oral cancer detection.

## In Brief

This study evaluated 430 referral letters of cases transferred to a Brazilian public oral medicine service and found frequent omission of essential clinical information, including lesion size, limits, symptoms, and evolution time. Incomplete documentation persisted, even in potentially malignant and malignant lesions, reinforcing the need for standardized referral templates.

## Graphical abstract

**Figure:**
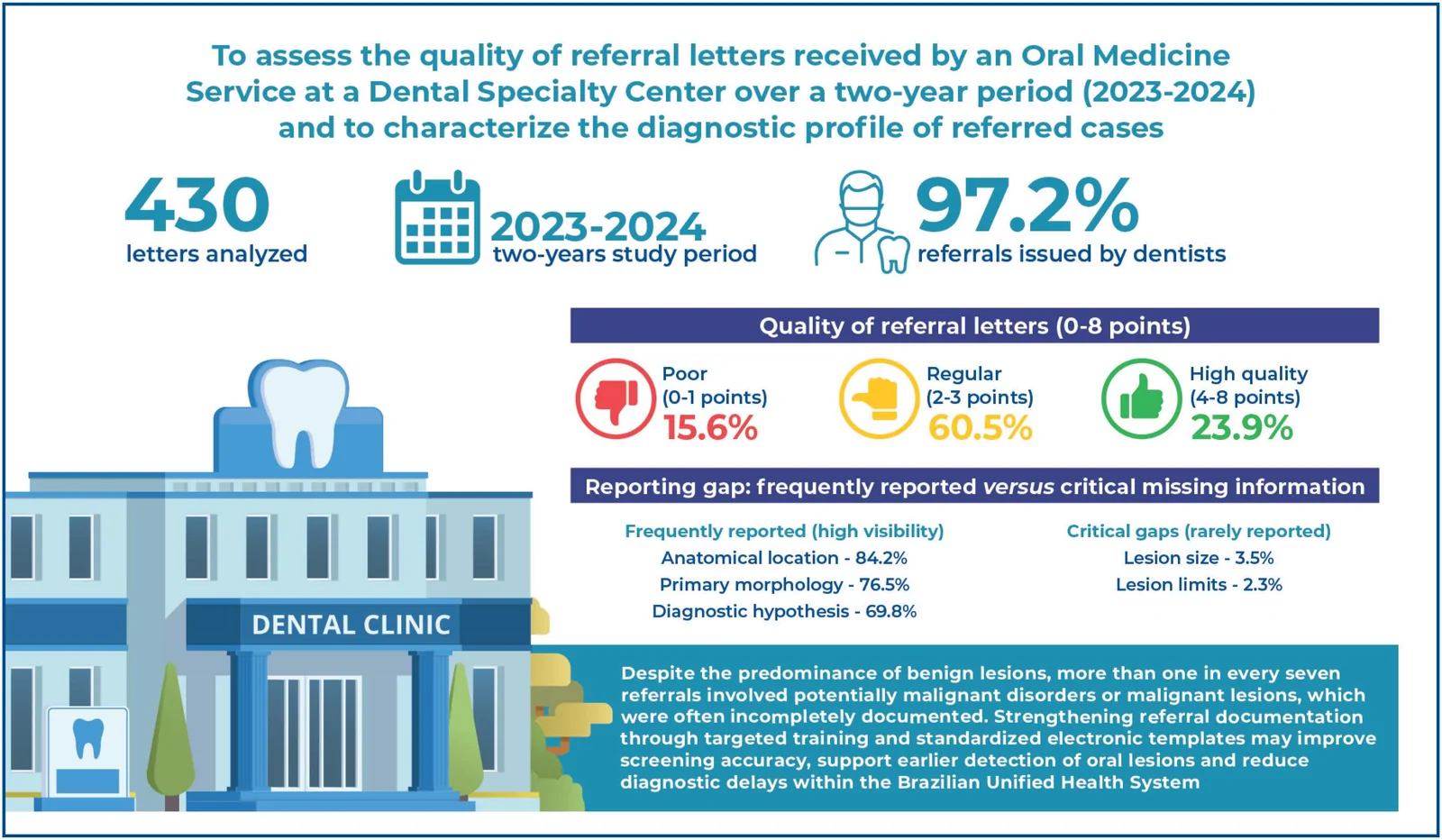

## INTRODUCTION

High-quality referral letters are essential for accurate diagnosis, effective screening, and timely management of oral lesions. Within Brazil's public health system (*Sistema Único de Saúde* [SUS]), access to specialized oral medicine services, primarily managed by public dental specialty centers (CEOs), mainly relies on the completeness of information provided by primary care professionals. As secondary-level referral units, CEOs depend on detailed clinical descriptions to appropriately prioritize oral potentially malignant disorders (OPMDs) and oral cancers.^(1)^

In the Brazilian public health system, referrals to oral medicine centers are typically issued by primary care units (*Unidade Básica de Saúde*), where general dentists and physicians perform initial clinical assessments. At this stage, the referral letter becomes the main source of information in arriving at a final diagnosis. The absence of essential descriptors, such as lesion morphology, precise anatomical location, duration of symptoms, symptomatology, or associated risk factors, may compromise case prioritization, delay access to specialized evaluation, and prevent the early detection of serious oral diseases.^(2,3)^

Evidence from different healthcare systems has consistently demonstrated that incomplete referral documentation disrupts communication across care levels and reduces diagnostic accuracy.^(4,5)^ Conversely, standardized and well-structured referrals enhance professional communication, optimize screening processes, and facilitate timely assessment of patients at high risk. In Brazil, structural challenges, such as regional inequalities in access to CEOs, shortages of trained Oral Medicine professionals, and heavy workloads in primary care settings, further worsen the adverse effects of poor-quality documentation of referrals.^(6,7)^ These systemic issues highlight the importance of strengthening referral practices to ensure continuity, safety, and efficiency across the oral healthcare network.

Several educational strategies and structured tools have been proposed to address this gap. Instruments such as OralDESC were developed to promote more standardized lesion descriptions in primary health care, while training initiatives on systematic oral examination have demonstrated improvements in lesion recognition skills among dental professionals.^(8,9)^ Despite these efforts, the consistency and completeness of referral letters within the public oral health network remain poorly explored.

Although previous Brazilian studies have examined the quality of referral letters in oral medicine, many have relied on single-center data, institution-specific contexts, or data collected prior to recent organizational and regulatory changes within the public health system.^(2,3)^ Moreover, few investigations have concurrently evaluated both referral quality and the diagnostic profile of referred lesions within contemporary regulatory frameworks. Therefore, updated analyses incorporating recent cohorts are essential to identify current deficiencies, refine screening, prioritize high-risk cases, and strengthen early detection pathways for oral cancer and OPMDs.

## OBJECTIVE

The present study aimed to assess the quality of referral letters received by an oral medicine service at a dental specialty center over a 2-year period (2023–2024) and to characterize the diagnostic profile of referred oral lesions.

## METHODS

This retrospective cross-sectional study evaluated the quality of referral letters registered at the oral medicine service of a dental specialty center (CEO) in Fernandópolis, São Paulo, Brazil, between January 1, 2023 and December 31, 2024. Ethical approval was obtained from the *Universidade Brasil* Research Ethics Committee (CAAE: 80863724.2.0000.5494; # 7.024.490).

All referral letters registered during the study period were included, characterizing this investigation as a census-based study rather than as selective sampling. Therefore, a priori sample size calculation was not applicable in this study.

A total of 430 eligible referral letters were retrieved from the institutional database. Extracted variables included patient demographic characteristics, referring professional category (dentist or physician), year of referral, and final diagnosis, established through clinical assessment and histopathological examination, when available.

The category "no diagnosis" was defined as cases without a definitive diagnosis after specialist evaluation, either due to inconclusive clinical findings, loss to follow-up, or insufficient diagnostic information, even after assessment.

### Assessment of referral letter quality

Each referral letter was assessed using eight predefined criteria organized into three domains: lesion description (primary morphology, anatomical location, lesion size, lesion limits, and consistency), presenting complaint history (symptomatology and time of evolution), and diagnostic hypothesis. The presence of each descriptor was determined according to standardized operational definitions to enhance reproducibility.

Primary morphology was deemed present when lesions were characterized using recognized clinical terms, such as macule, papule, plaque, ulcer, nodule, vesicle, or erosion. Anatomical location was recorded when the anatomical subsite was explicitly specified (e.g., buccal mucosa, tongue border, and palate). Lesion size was considered present when any numerical measurement was documented in millimeters or centimeters. Lesion limits were recorded when borders were described as well-defined, ill-defined, regular, or irregular. Consistency was classified as present when tactile descriptors such as soft, firm, indurated, or fluctuant were reported.

Symptomatology was considered present when patient complaints, including pain, burning sensation, bleeding, or functional impairment, were documented. The time of evolution was recorded when the lesion duration was specified in days, weeks, months, or years. A diagnostic hypothesis was registered whenever an explicit clinical impression or differential diagnosis was reported.

Each present criterion scored 1 point, generating a total score ranging from 0 to 8. Following the classification previously proposed by Rodrigues et al.^(3)^ referral letters were categorized as poor (0–1 points), regular (2–3 points), good (4–6 points), or excellent (7–8 points). Although this scoring system has been previously used in Brazilian studies, its psychometric properties, such as construct validity and reliability, has not been formally validated. Nevertheless, it was considered appropriate for the present study due to its structured, objective, and clinically relevant criteria for assessing referral documentation.

### Evaluator characteristics

All referral letters were evaluated by a single examiner, an oral medicine specialist with experience in referral documentation analysis. To enhance consistency, a pilot calibration was conducted prior to the main analysis. During this phase, a subset of referral letters was independently reviewed and discussed among the authors until consensus regarding the interpretation of each criterion was achieved.

No formal intra-examiner reliability testing (e.g., test-retest) was performed. Therefore, although consistency was ensured through calibration, some degree of subjective interpretation, particularly in borderline classifications (e.g., between "regular" and "good"), cannot be excluded.

### Inclusion and exclusion criteria

Only first-time referrals submitted for diagnostic purposes were included. Letters referring patients for routine dental procedures or cases with previously confirmed diagnoses were excluded from the analyses.

### Statistical analyses

Descriptive statistics (absolute and relative frequencies) were calculated using Microsoft Excel (Microsoft Corporation, Redmond, WA, USA). The Kruskal–Wallis test, followed by Mann-Whitney pairwise comparison with Bonferroni adjustment, was used to compare the scores across the diagnostic categories. P values <0.05 were considered statistically significant. Analyses and graph construction were performed in Python (Python Software Foundation, Wilmington, Delaware, United States) using the packages *pandas* for data handling, *SciPy* for statistical testing, and *matplotlib* for data visualization.

## RESULTS

A total of 430 referral letters met the inclusion criteria. All referrals were from primary public healthcare services, with 202 (47.0%) issued in 2023 and 228 (53.0%) in 2024. Most referrals were made to dentists (418; 97.2%), while referrals to physicians accounted for only 12 cases (2.8%).

Regarding overall quality, 260 referral letters were classified as regular (60.5%), followed by 99 good (23.0%) and 67 poor (15.6%); only 4 letters (0.9%) met the criteria for excellent quality (Table 1). Quality distributions were similar between the professional categories, with no reasonable differences between dentists and physicians (Table 2). This finding indicates that incomplete documentation is a systemic issue rather than a profession-specific trend.

**Table 1:** Classification of referral letters according to quality

Classification	n (%)
Poor	67 (15.6)
Regular	260 (60.5)
Good	99 (23.0)
Excellent	4 (0.9)
Total	430 (100)

**Table 2:** Classification of referral letters according to profession

Professional	Classification				Total
	Poor	Regular	Good	Excellent	
Dentist	66	252	96	4	418
Physician	1	8	3	0	12
Total	67	260	99	4	430

Analysis of individual descriptors revealed a marked imbalance between frequently reported and poorly documented clinical criteria. Although most letters described lesion location (84.2%) and primary morphology (76.5%), fewer than one quarter of the letters included symptomatology (22.8%) or time of evolution (16.0%). Clinically essential descriptors of malignancy risk assessment were rarely documented: lesion size (3.5%) and lesion limits (2.3%) were the least reported variables, followed by consistency (6.5%). These findings highlight substantial gaps in fundamental clinical characterization required for adequate risk stratification (Table 3).

**Table 3:** Predefined criteria in referral letters

Criteria	n (%)
Location	362 (84.2)
Primary morphology	329 (76.5)
Diagnostic hypothesis	300 (69.8)
Symptomatology	98 (22.8)
Time of evolution	69 (16.0)
Consistency	28 (6.5)
Size	15 (3.5)
Limits	10 (2.3)

Final diagnoses were predominantly benign lesions (309; 71.9%), followed by OPMDs (46; 10.7%), no diagnosis (32; 7.4%), normality variations (27; 6.3%), and malignant lesions (16; 3.7%) (Table 4). Importantly, referral quality scores did not differ substantially between benign and high-risk conditions (OPMDs or malignant lesions). Thus, referrals for a potentially serious disease were frequently characterized by incomplete clinical documentation, which may potentially compromise prioritization processes and contribute to delays in diagnostic pathways.

**Table 4:** Classification of referral letters according to diagnostic category

Diagnostic category	n (%)
Benign lesions	309 (71.9)
OPMDs	46 (10.7)
No diagnosis	32 (7.4)
Normality variations	27 (6.3)
Malignant lesions	16 (3.7)
Total	430 (100)

OPMDs: oral potentially malignant disorders.

A significant difference in score distributions among diagnostic categories was observed (H(4)=13.92, p=0.0076), with a small effect size (η²≈0.03). Median scores were lower in cases without a final diagnosis than in benign lesions, reinforcing the association between incomplete documentation and diagnostic uncertainty. Post-hoc Mann–Whitney tests with Bonferroni correction showed that "no diagnosis" cases had significantly lower scores than those with benign lesions (p=0.003). No other pairwise comparisons reached statistical significance (p>0.05) (Figure 1).

**Figure 1:**
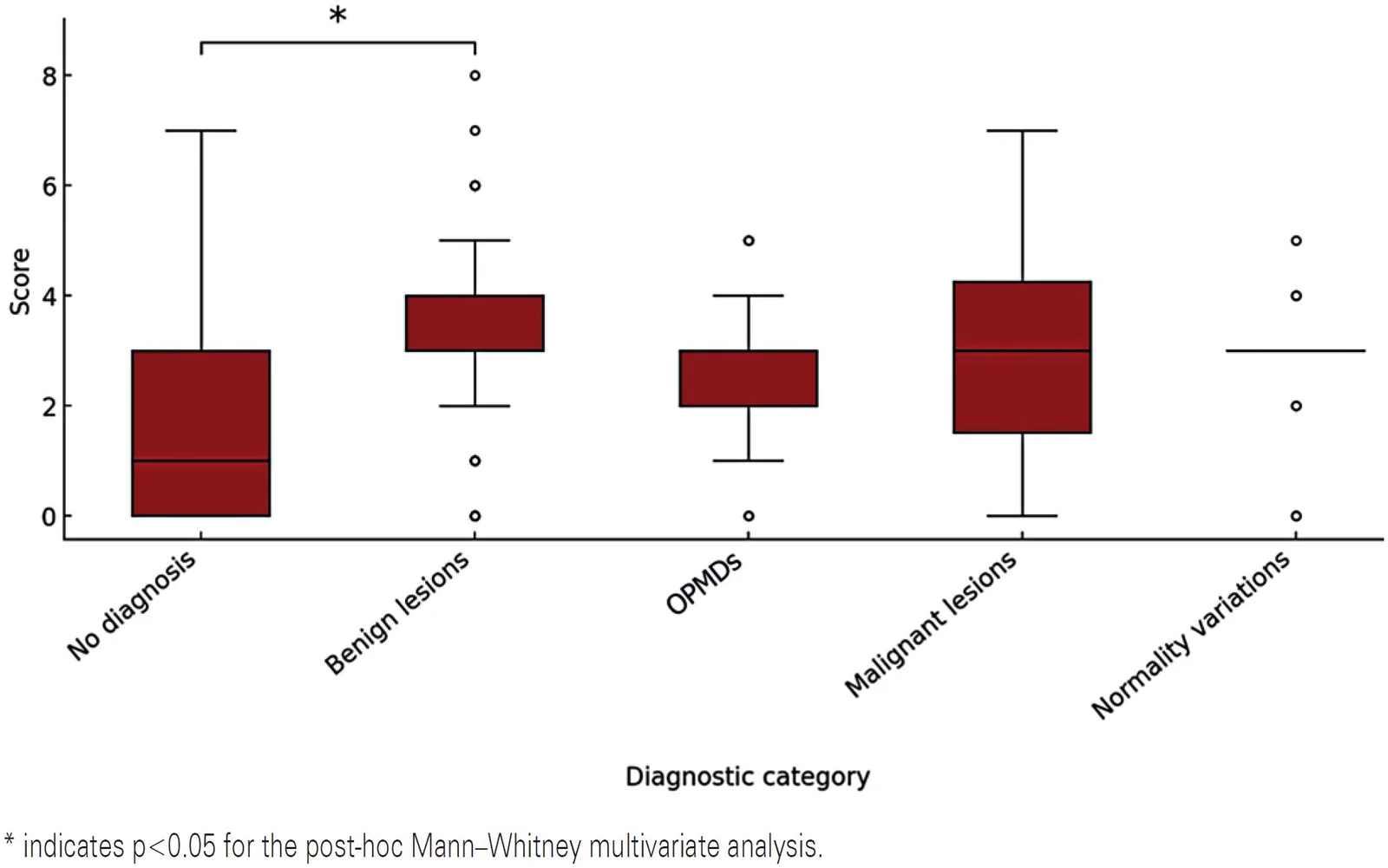
Distribution of referral quality scores according to diagnostic category

## DISCUSSION

This study provides one of the most recent evaluations (2023–2024) of referral letters to specialized oral medicine care quality within Brazil's public oral health network; it is among the few investigations to concurrently analyze documentation completeness alongside diagnostic outcomes, including OPMDs and malignant lesions. Although most letters were classified as regular or good, approximately three-quarters of the referrals still fell within low-quality categories (poor or regular). This finding is consistent with those of previous Brazilian studies^(2,3)^ and comparable to international findings on inadequate clinical communication.^(4)^ Oral medicine specialists have previously reported that general dental practitioners often fail to provide details for clinical features of oral lesions and do not report patients’ medical history on referral letters.^(10)^ In this context, any apparent improvement relative to historical data remains modest and insufficient to guarantee safe and efficient screening pathways.

As all referrals received over the 2-year period were included, this study represents a census of service activity, minimizing selection bias and strengthening internal validity. Nevertheless, as it is a single-center study, generalization to other Brazilian regions with different healthcare structures should be done cautiously.

Our descriptive findings did not indicate marked differences in referral quality according to year of attendance or professional category. This suggests that inadequate documentation may reflect a broader pattern of practice rather than deficiencies restricted to specific professional groups or short-term organizational fluctuations. This finding reinforces the interpretation that current referral deficiencies are primarily rooted in structural and educational gaps rather than individual professional performance alone.

From a clinical perspective, the magnitude of missing information in key descriptors warrants particular attention. Essential variables for diagnostic risk stratification, such as lesion size, lesion limits, consistency, symptomatology, and time of evolution, were absent in most referrals. These proportions mirror those reported by Navarro et al.^(2,11)^ and Rodrigues et al.,^(3)^ indicating that despite advances in training initiatives over the past two decades, progression in routine documentation practices is limited. Furthermore, poor clinical descriptions may have an impact beyond interprofessional communication, as reported in a recent study that general dental practitioners fail to provide complete clinical and radiographic information when consulting ChatGPT for diagnostic hypothesis in oral Medicine cases.^(12)^ Similarly, an international study reported that incomplete referrals compromise diagnostic accuracy and delay care pathways^(4)^ suggesting that this challenge extends beyond local contexts and reflects a broader issue in healthcare communication.

An important contribution of this study is the observation that the inclusion of symptomatology, evolution time, and lesion size was strongly associated with higher referral quality scores, validating the clinical relevance of the scoring system. These descriptors directly influence malignancy risk assessment and urgency classification, and their frequent omission from referral letters significantly undermines the screening function of referral documentation. Clinically, this limitation extends beyond suboptimal prioritization, potentially resulting in delayed specialist evaluation, biopsy, and definitive diagnosis in patients with potentially malignant or malignant lesions.

This concern is further reinforced by the finding that referral quality for high-risk cases (OPMDs and malignant lesions) was not superior to that observed for benign conditions. This lack of differentiation is a clinically significant gap, as cases requiring faster assessment were not accompanied by more detailed or higher-quality documentation. Evidence from the literature has consistently indicated that inadequate referral documentation is associated with delays in diagnostic pathways.^(5)^ This suggests that deficiencies in communication between primary care and specialized services may contribute to the delayed evaluation of patients with potentially malignant or malignant lesions, although this relationship was not directly assessed in the present study.

Structural characteristics of the Brazilian public health system compound these challenges. The unequal geographic distribution of CEOs, high service demand, variable waiting times, and shortages of trained oral medicine specialists create bottlenecks across the care network.^(6,7,13)^ In such an overloaded system, incomplete referral documentation increases the burden on specialists, who spend additional consultation time obtaining clinical information that should have been recorded at the primary care level. This inefficiency reduces service throughput and may indirectly contribute to prolonged diagnostic waiting periods.

Therefore, educational and organizational strategies to improve referral quality remain highly relevant. Tools such as OralDESC have been designed to systematize lesion description in primary care, and training initiatives focused on comprehensive oral examination have demonstrated improvements in lesion detection and professional communication.^(8,9)^ However, the persistent shortcomings identified in this study suggest that these approaches have not yet been sufficiently integrated into routine practice, underscoring the need for broader institutional adoption and continuous professional education.

Telehealth initiatives represent a complementary strategy to address these gaps. Programs such as EstomatoNet have shown potential in enhancing diagnostic communication and reducing unnecessary referrals.^(14)^ Regarding the frequent documentation lapses observed in this study, telehealth platforms may offer real-time support to primary care professionals, not only as diagnostic consultation tools but also as mechanisms to promote compliance with minimum documentation standards before preparing a referral letter.

The diagnostic profile observed reinforces the importance of strengthening the quality of referral documentation. Although benign lesions predominated, the detection of OPMDs and malignant conditions confirms that primary care professionals generally demonstrate adequate initial recognition of suspicious lesions. The primary challenge appears less related to case identification and more related to the translation of clinical suspicion into systematic, comprehensive referral documentation that can support effective risk stratification and timely diagnostic action.

Beyond its academic value, these findings reinforce the need for the immediate implementation of standardized electronic referral templates within the SUS, particularly for high-risk oral cancer and OPMDs referral pathways. Developing structured referral forms that incorporate mandatory fields for lesion size, limits, symptomatology, consistency, and evolution time can directly address the less frequently reported descriptors identified in this study, enabling more accurate prioritization by specialized services. Such standardization can optimize clinical decision-making, reduce diagnostic delays, and improve the overall efficiency and safety of the oral cancer care network.

From a public health policy perspective, these results align with ongoing national initiatives in Brazil to strengthen digital health infrastructure, including the expansion of the e-SUS primary care system and improvements in the regulatory management of referral flows across regional care networks. Integrating standardized electronic referral templates into clinical information systems could establish minimum quality standards for inter-level communication, enhance regulatory screening processes, and generate structured datasets to support epidemiological surveillance and continuous service evaluation. This approach directly supports SUS's strategic priorities related to care coordination, patient safety, and early oral cancer detection.

This study has important strengths, including its large sample size, use of structured scoring criteria that have been used in previous studies, and focus on a contemporary cohort within a regulated public health system. Nonetheless, some limitations should be acknowledged. Regarding methodological limitations, referral letters were evaluated by a single examiner, and inter-rater reliability was not assessed. This may have introduced a degree of subjective interpretation. Although a prior calibration process was conducted, no formal intra-examiner reliability assessment was performed and some variability in borderline classifications (e.g., between "regular" and "good") cannot be excluded. Future studies should include independent evaluators and report reliability coefficients (e.g., Cohen's kappa or intraclass correlation coefficients) to strengthen methodological robustness. Furthermore, besides the single-center design, data analysis was restricted to written referral documentation and did not permit the evaluation of downstream outcomes, such as waiting times for specialist assessment, biopsy intervals, or cancer stage at diagnosis.

In summary, the persistent incompleteness of referral letters highlights a critical communication gap between primary care services and specialized oral medicine centers within the SUS. Expanding standardized documentation tools, strengthening professional training initiatives, and integrating telehealth support systems may significantly enhance screening quality, reduce diagnostic delays, and ultimately improve patient outcomes, particularly among individuals with OPMDs or oral cancer.

## CONCLUSION

This study showed that although most referred lesions were benign, referral letters predominantly had low to moderate quality and frequently lacked key clinical descriptors, regardless of the oral lesion type. Despite the predominance of benign conditions, the statistically confirmed low overall quality of the referral letters highlights persistent communication gaps between primary care and specialized oral medicine services. More than 14% of referred cases involved oral potentially malignant disorders or malignant lesions; therefore, incomplete documentation may compromise the timely identification and prioritization of patients at high risk. Strengthening referral quality through standardized templates, structured training programs (including simulation-based education), and referral tools integration into electronic health systems can improve screening efficiency, support the earlier detection of oral potentially malignant disorders and oral cancer, and optimize oral medicine referral pathways within the Brazilian Brazil's public health system. Furthermore, the strong associations observed between high-quality referrals and the presence of essential clinical descriptors validate the scoring instrument used and reinforce the clinical relevance of standardized documentation.

## Data Availability

The underlying content is contained within the manuscript.
